# 

**Published:** 1797

**Authors:** 


### CONTENTS.

Page

I. *PRACTICAL Observations on the Treatment of acute Diseases; particularly those of the West Indies. By* William Wright, *M. D. Fellow of the Royal College of Physicians of Edinburgh, and of the Royal Societies of London and Edinburgh; and Physician to the Forces in the West Indies.* — — 1

II. *Facts relative to the Origin of intermittent Fevers. By* Thomas Beddoes, *M. D.* 26

III. *Observations on the Nature of Corns, and the Means of removing them. By Mr.* Anthony Carlisle, *Surgeon to the Westminster Hospital.* — — 29

IV. *Some Observations relative to the Angustura Bark. By* Thomas Masterman Winterbottom, *M. D. Physician to the Settlement at Sierra Leone.* — 41

V. *An Account of a remarkable Affection of the Testes. By Mr.* Widdows Golding, *Surgeon at Wallingford, in Berkshire, and*

2 *Member*

Page

*Member of the Corporation of Surgeons in London.* — — 62

VI. *Case of a Man who castrated himself. By the same.* — — 74

VII. *Cases and Remarks on the external Application of Charcoal; by Mr.* William Simmons, *Member of the Corporation of Surgeons in London; and Surgeon to the Manchester Infirmary.* — 77

VIII. *Case of Pins extracted from the Breast of a Woman, after remaining there sixty Years. By Mr.* Henry Fryer, *Surgeon at Stamford, in Lincolnshire. Communicated in a Letter to Dr.* Simmons, *by* John Clarke, *M. D. Physician in London.* 86

IX. *Description of a new Key Instrument for the Extraction of Teeth. By Mr.* J. Savigny, *Surgical-Instrument Maker in London.* — — 90

X. *Some Account of the Effects of the Vapour of Vitriolic Æther in Cases of Phthisis Pulmonalis. By* Richard Pearson, *M. D. Member of the Royal College of Physicians, London; and Physician to the General Hospital near Birmingham.* — 95

XI. *Account of two Instances of uncommon Formation*

Page

*mation in the Viſcera of the Human Body. By Mr.* John Abernethy, *Aſſiſtant Surgeon to St. Bartholomew's Hoſpital. From the Philoſophical Tranſactions of the Royal Society of London.* —— 100

XII. *Deſcription of an extraordinary Production of Human Generation; with Obſervations. By* John Clarke, *M. D. From the ſame Work.* — — 109

XIII. *On the Converſion of Animal Muſcle into a Subſtance much reſembling Spermaceti. By* George Smith Gibbes, *B. A. of Magdalen College, Oxford. From the ſame Work.* — — 121

XIV. *Experiments on the Nerves, particularly on their Reproduction; and on the ſpinal Marrow of living Animals. By* William Cruikſhank, *Eſq. From the ſame Work.* 136

XV. *An experimental Inquiry concerning the Reproduction of Nerves. By* John Haighton, *M. D. From the ſame Work.* 155

XVI. *Deſcription of a Human Male Monſter, with Remarks. By* Alexander Monro, *M. D. F. R. S. Edin. Fellow of the Royal College of Phyſicians, Profeſſor of Medicine, Anatomy, and Surgery in the Univerſity of Edin-*

Page

*Edinburgh, Fellow of the Royal Academy of Surgery in Paris,* &c. *From the Transactions of the Royal Society of Edinburgh.* 170

XVII. *Description of an Instrument for performing the Operation of trepanning the Skull, with more Ease, Safety, and Expedition than with those now in general Use. By* Samuel Croker King, *Esq. Member of the Royal College of Surgeons in Ireland, and M. R. I. A. From the Transactions of the Royal Irish Academy.* — 191

XVIII. *Case of an Enlarged Spleen. By* George Burrowes, *M. D. M. R. I. A. From the same Work.* — 219

XIX. *An Estimate of the Excess of the Heat and Cold of the American Atmosphere beyond the European, in the same Parallel of Latitude: to which are added, some Thoughts on the Causes of this Excess. By* Edward Augustus Holyoke, *M. D. F. A. A. From the Memoirs of the American Academy of Arts and Sciences.* — — 225

XX. *An Account of an uncommon Case of Emphysema; and of an external Abscess, the Contents of which were discharged by coughing. By the same. From the same Work.* 259

XXI.

Page

XXI. *Account of a Locked Jaw. By* Aaron Dexter, *M. D. F. A. A. From the ſame Work.* — — 266

XXII. *An Account of the Effects of Negative Electricity, in Caſes of Burns. By Mr.* John Vinall. *From the ſame Work.* 279

XXIII. *Deſcription of a Caſe of Hydrocephalus. By M.* Tenghil, *Profeſſor of Surgery at Quiers. From the Memoirs of the Royal Academy of Sciences at Turin.* — 281

XXIV. *Account of a Caſe in which a Stone, formed in one of the Kidneys, was extracted through an Abſceſs in the Back. By* Herman Schützercrants, *M. D. From the Tranſactions of the Royal Academy of Sciences at Stockholm.* — 285

XXV. *An Account of the poiſonous Quality of the Juice of the Root of Jatropha Manihot, or bitter Caſſada; and of the Uſe of Cayenne Pepper in counteracting the Effects of this and ſome other poiſonous Snbſtances; with Remarks on the Efficacy of the Spigelia Anthelmia in Worm Caſes. By* James Clark, *M. D. Phyſician in Dominica, and Fellow of the College of Phyſicians and Royal Society of Edinburgh.* — — 289

XXVI.

Page

XXVI. *An Account of ſome Experiments made with a View to aſcertain the comparative Quantities of amylaceous Matter, yielded by the different Vegetables moſt commonly in uſe in the Weſt India Iſlands. By the ſame.* 300

XXVII. *A fatal Inſtance of the poiſonous Effects of the Oenanthe Crocata* Linn. *or Hemlock Dropwort. By* Robert Graves, *M. D. Phyſician at Dorcheſter, and Extra Licentiate of the College of Phyſicians, London.* — — 308

*Catalogue of Books.* — — 313

*Index.* — — — 372

